# Total mesorectal excision in MRI-defined low rectal cancer: multicentre study comparing oncological outcomes of robotic, laparoscopic and transanal total mesorectal excision in high-volume centres

**DOI:** 10.1093/bjsopen/zrae029

**Published:** 2024-05-24

**Authors:** Marieke L Rutgers, Thijs A Burghgraef, Jeroen C Hol, Rogier M Crolla, Nanette A van Geloven, Jeroen W Leijtens, Fatih Polat, Apollo Pronk, Anke B Smits, Jurriaan B Tuyman, Emiel G Verdaasdonk, Colin Sietses, Esther C Consten, Roel Hompes

**Affiliations:** Department of Surgery, Amsterdam University Medical Centre, Amsterdam, The Netherlands; Department of Surgery, Meander Medical Centre, Amersfoort, The Netherlands; Department of Surgery, University Medical Centre, Groningen, The Netherlands; Department of Surgery, Amsterdam University Medical Centre, Amsterdam, The Netherlands; Department of Surgery, Hospital Gelderse Vallei, Ede, The Netherlands; Department of Surgery, Amphia Hospital, Breda, The Netherlands; Department of Surgery, Tergooi Hospital, Hilversum, The Netherlands; Department of Surgery, Laurentius Hospital, Roermond, The Netherlands; Department of Surgery, Canisius Wilhelmina Hospital, Nijmegen, The Netherlands; Department of Surgery, Diakonessenhuis, Utrecht, The Netherlands; Department of Surgery, St. Antonius Hospital, Nieuwegein, The Netherlands; Department of Surgery, Amsterdam University Medical Centre, Amsterdam, The Netherlands; Department of Surgery, Jeroen Bosch Hospital, Den Bosch, The Netherlands; Department of Surgery, Hospital Gelderse Vallei, Ede, The Netherlands; Department of Surgery, Meander Medical Centre, Amersfoort, The Netherlands; Department of Surgery, University Medical Centre, Groningen, The Netherlands; Department of Surgery, Amsterdam University Medical Centre, Amsterdam, The Netherlands

## Abstract

**Background:**

The routine use of MRI in rectal cancer treatment allows the use of a strict definition for low rectal cancer. This study aimed to compare minimally invasive total mesorectal excision in MRI-defined low rectal cancer in expert laparoscopic, transanal and robotic high-volume centres.

**Methods:**

All MRI-defined low rectal cancer operated on between 2015 and 2017 in 11 Dutch centres were included. Primary outcomes were: R1 rate, total mesorectal excision quality and 3-year local recurrence and survivals (overall and disease free). Secondary outcomes included conversion rate, complications and whether there was a perioperative change in the preoperative treatment plan.

**Results:**

Of 1071 eligible rectal cancers, 633 patients with low rectal cancer were identified. Quality of the total mesorectal excision specimen (*P* = 0.337), R1 rate (*P* = 0.107), conversion (*P* = 0.344), anastomotic leakage rate (*P* = 0.942), local recurrence (*P* = 0.809), overall survival (*P* = 0.436) and disease-free survival (*P* = 0.347) were comparable among the centres. The laparoscopic centre group had the highest rate of perioperative change in the preoperative treatment plan (10.4%), compared with robotic expert centres (5.2%) and transanal centres (2.1%), *P* = 0.004. The main reason for this change was stapling difficulty (43%), followed by low tumour location (29%). Multivariable analysis showed that laparoscopic surgery was the only independent risk factor for a change in the preoperative planned procedure, *P* = 0.024.

**Conclusion:**

Centres with expertise in all three minimally invasive total mesorectal excision techniques can achieve good oncological resection in the treatment of MRI-defined low rectal cancer. However, compared with robotic expert centres and transanal centres, patients treated in laparoscopic centres have an increased risk of a change in the preoperative intended procedure due to technical limitations.

## Teaser

This multicentre, retrospective cohort study showed that with all three minimally invasive techniques (robot, transanal and laparoscopy) good oncological resection can be achieved in the treatment of MRI-defined low rectal cancer. However, patients treated in laparoscopic centres have an increased risk of a change in the preoperative intended procedure due to technical limitations.

## Introduction

Surgical resection according to the total mesorectal excision (TME) principle remains one of the key elements in the treatment of rectal cancer^1,2^. Robot-assisted surgery (R-TME) and transanal surgery (TaTME) may overcome some of the challenges found in laparoscopic rectal cancer surgery (L-TME) due to enhanced visualization of and access to embryological planes deep in the pelvis. Especially in LOw REctal Cancer (LOREC), this could lead to a superior TME specimen with less positive resection margins (R1), lower conversion rates and more primary anastomoses^3–7^. Nevertheless, current literature does not usually describe outcomes for LOREC separately.

The most appropriate definition for LOREC is still debated, but is mainly based on tumour height from the anal verge or anal ring^8^. However, the use of tumour height may lack precision due to variations in individual anatomy^9–12^. An MRI-based definition of LOREC offers a more standardized approach and allows for the identification of technically challenging cases. According to the UK LOREC programme, a low rectal tumour can be identified through the anatomical landmark where the tapering of the mesorectum starts, which can be seen on sagittal MRI^8,13–15^. So far, this new definition of LOREC is only utilized in a handful of papers, with very little actual clinical data. Furthermore, most published data on R-TME and TaTME behold results of cases performed during the learning curve which may not fully represent the true potential of these techniques^16,17^. For TaTME, the learning curve is set at around 45–51 cases, followed with a minimum caseload of 25–30 cases per year to ensure expertise and procedural quality^18,19^. The learning curve for robotic TME appears to be similar^20,21^.

The present study aimed to compare the oncological outcomes of MRI-defined LOREC in centres that completed the learning curve in different minimally invasive techniques. Secondary outcome measures include conversion rates, perioperative complications and changes in the preoperative surgical plan.

## Methods

A retrospective, multicentre cohort study was conducted in 11 centres in The Netherlands with large experience in one of the three minimally invasive techniques (three TaTME, three R-TME and five L-TME centres). The study protocol was approved by the Medical Research Ethics Committees United (MEC-U, AW 19.023/W18.100) and the local ethical boards of all participating centres.

### Data extraction

Data were initially obtained from the Dutch Colorectal Audit (DCRA), which is a mandatory nationwide registry that collects information on all surgically resected primary colorectal cancer patients. Missing data and additional information not available in the DCRA data set were added by local investigators with use of the local electronic medical records. Data extracted consisted of outpatient visit notes, multidisciplinary team (MDT) notes, surgical notes, radiology reports and images, pathology reports, (neo)adjuvant therapy details and complications. All pseudonymized data were collected between January and April 2020 in the data management system CASTOR, a research data collector system^22^.

### Patient selection

All patients 18 years and older, diagnosed with rectal cancer, adhering to the sigmoidal take-off definition^14^, for which they underwent rectal cancer surgery (open or minimally invasive) with curative intent between January 2015 and December 2017 in 1 of the 11 participating hospitals were included. The groups were divided according to the minimally invasive technique used in the respective centres. To participate, each centre had to perform a minimum of 30 procedures per year using the expert technique. Procedures performed in the year 2015 at two centres where the expert technique was introduced in 2014 (one robot and one TaTME centre respectively) were excluded because it was assumed that for those cases the learning curve had not fully run its course.

Subsequently all staging MRI scans were reviewed by trained local investigators to identify the cohort of patients with MRI-defined LOREC according to the defined criteria^8,15,21^. Excluded from analysis were patients with palliative treatment, synchronous colonic tumours, acute procedures, hyperthermic intraperitoneal chemotherapy (HIPEC), intraoperative radiotherapy (IORT) and non-TME surgery (including local excision, transanal endoscopic microsurgery (TEM) or transanal minimally invasive surgery (TAMIS)). Open surgery procedures were not excluded, but since seldom performed, were not analysed in depth for the outcomes of interest (see below). Each patient was discussed by a local MDT and indications for neoadjuvant treatment were given according to the current Dutch national guidelines for colorectal cancer.

### Outcomes of interest and definitions

Outcomes of interest were compared among the centres (L-: laparoscopic TME centres, R-: robotics TME centres and Ta-: TaTME centres). The primary outcomes were the oncological outcomes, specifically the rate of positive resection margins (R1) and quality of the TME specimen, and 3-year local recurrence, distant recurrence, overall survival and disease-free survival. Secondary outcomes included conversion rate, intraoperative changes to the procedural plan, incidence of restorative procedures (with or without diverting stoma), and the intra- and postoperative outcomes (for example 30-day morbidity rate, anastomotic leakage rate (AL) and 30-day readmission rate).

A LOREC tumour was defined as a tumour with the lower border located distal to the point where the levator ani muscles insert on the pelvic bone, as identified on sagittal MRI^8,15,21^. The TaTME technique combines an endoscopic transanal approach with a laparoscopic abdominal approach. R-TME is a procedure where (at least) the pelvic dissection was performed with the da Vinci surgical system.

The type of procedure was categorized as either low anterior resection (LAR) with anastomosis, encompassing all sphincter-saving procedures with primary anastomosis with or without a temporary stoma. Non-restorative procedures included LAR with end colostomy (Hartmann’s procedure) or abdominoperineal resection (APR), which includes any procedure with perineal dissection with complete, intersphincteric, or extrasphincteric proctectomy and definitive end colostomy.

To avoid selection bias, other techniques used to treat low rectal tumours in a specific expert centre, such as the use of laparoscopy for APR in TaTME and R-TME centres, were not excluded from analysis.

A positive mesorectal fascia (MRF) was defined as a distance ≤1 mm from the resection margin on MRI. Circumferential and distal resection margin involvement (CRM+ and DRM+ respectively) were defined as a tumour or malignant lymph node ≤1 mm from the circumferential or distal resection margin, and were both regarded as R1 resections. A conversion to open surgery was defined as the use of a median laparotomy or any accessory incision for purposes other than specimen extraction. The 30-day morbidity rate was categorized according to the Clavien–Dindo classification^23^. Anastomotic leakage was defined as anastomotic dehiscence or intra-abdominal abscess adjacent to the anastomotic site, including those occurring beyond 30 days. The leakages were graded according to the International Study Group of Rectal Cancer (ISREC) classification for anastomotic leakage after anterior resection, where grade A can be managed without changing the management, grade B requires active therapeutic intervention but without reoperation on and grade C requires laparotomy^24^. Change in the preoperative treatment plan was defined as a change in the operative plan agreed by the MDT (i.e., from a restorative to a non-restorative procedure). Information was extracted from the surgical notes. A secondary stoma was defined as a second stoma formation after a first attempt of primary stoma reversal.

### Statistical analysis

Descriptive statistics were used to describe baseline characteristics. Categorical variables were presented as numbers and percentages, while continuous variables were presented as mean(s.d.) or median (interquartile range) depending on the type of distribution. Univariable analysis was performed using the chi-square test for categorical data. The independent sample *t*-test or the Wilcoxon-rank sum test were used for continuous data. Multivariable logistic regression analysis, using backward selection, was conducted to evaluate independent factors associated with CRM positivity, anastomotic leakage and a change in procedural plan. Variables taken into account were: actual surgical technique (L-TME, R-TME and TaTME), age, BMI, sex, ASA score, surgical abdominal history, distance to the anorectal junction (ARJ), MRF, clinical tumour (cT) stage, clinical node (cN) stage, clinical metastasis (cM) stage and neoadjuvant therapy. The results were expressed as odds ratios (OR) with 95% c.i. A *P* value of <0.05 was considered significant. Statistical analysis was performed using R, version 3.6.2 (R Foundation for Statistical Computing, Vienna, Austria).

## Results

**Fig. 1:**
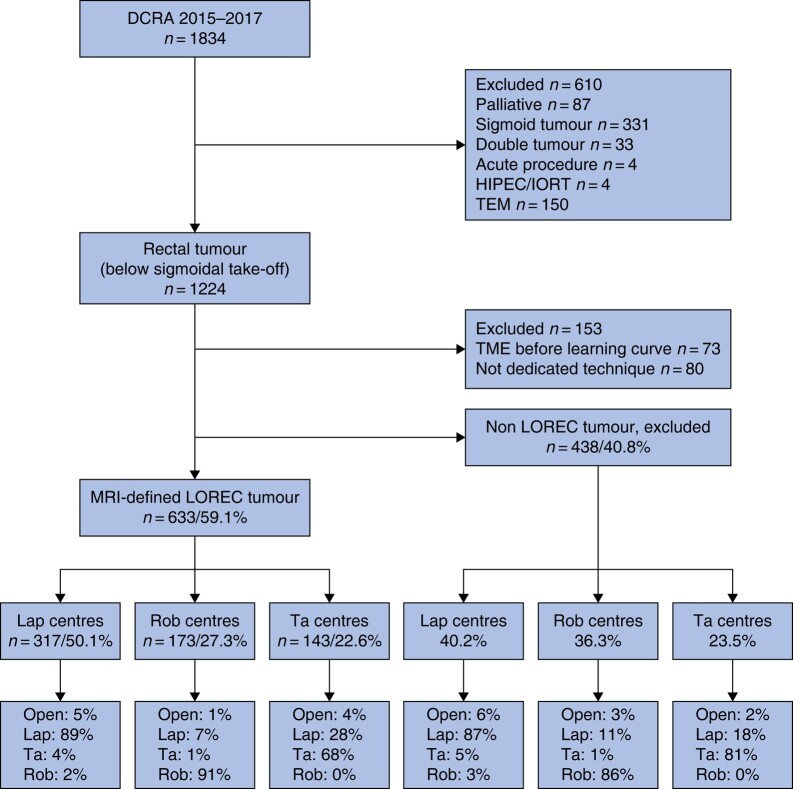
Flow diagram of included patients

DCRA, Dutch Colorectal Audit; TME, total mesorectal excision; LOREC, LOw REctal Cancer; Lap, laparoscopic; Rob, robot assisted; Ta, transanal TME; HIPEC, hyperthermal intraperitoneal chemotherapy; IORT, intraoperative radiotherapy; TEM, transanal endoscopic microsurgery.

A total of 1834 patients with rectal cancer were registered in the DCRA registry in the participating centres between January 2015 and December 2017. After excluding patients that did not meet the inclusion criteria and/or were located above the sigmoidal take-off, 1071 patients with rectal cancer remained. Among them 633 (59.1%) were classified as MRI-defined LOREC tumours (*Fig. 1*).

### Baseline characteristics

Baseline patient and tumour demographics are displayed in *Table 1*. Patients from Ta-centres were younger, with a mean age of 65 years, compared with 67 years in L-centres and 68 years in R-centres, *P* = 0.04. Ta-centre patients had a shorter distance to the anorectal junction (ARJ), with a mean distance of 2 cm, compared with 3 cm in both L- and R-centres, *P* = <0.001. Additionally, Ta-centres had the highest proportion of patients with cN0 stage (52%), compared with 40% in L- and 36% in R-centres, *P* = 0.01. Patients in R-centres received the most neoadjuvant therapy (72%) compared with L-centres (65%) and Ta-centres (66%), *P* = 0.027.

**Table 1:** Baseline characteristics MRI-defined LOREC patients, 2015–2017

Patient characteristics		LOREC				
		Total *n* = 633 (%)	L-centre *n* = 317	R-centre *n* = 173	Ta-centre *n* = 143	*P* value
Age (years), mean(s.d.)		67(10.5)	67(9.9)	68(10.6)	65(11.7)	0.038
BMI (kg/m^2^), mean(s.d.)		26(4.3)	26(4.4)	26(4.0)	26(3.9)	0.475
**Sex**						
Male		63.5	197 (62.1)	109 (63.6)	95 (66.4)	0.676
Female		36.5	120 (37.9)	63 (36.6)	48 (33.6)	
**ASA**						
I		19.7	55 (17.4)	40 (23.1)	30 (21.0)	0.286
II		60.3	200 (63.1)	93 (53.8)	89 (62.2)	
III		19.0	57 (18.0)	40 (23.1)	23 (16.1)	
IV		0.9	5 (1.6)	0 (0.0)	1 (0.7)	
History of abdominal surgery	Yes	30.0	98 (30.9)	48 (27.7)	44 (30.8)	0.746
Distance of tumour to ARJ (cm), median (i.q.r.)		3 (0–5)	3 (1–5)	3 (1–5)	2 (0–3)	<0.001
Preoperative MRF	≤1 mm	34.5	97 (30.9)	67 (39.0)	53 (37.1)	0.129
**cT-stage**						
cT1		0.2	0 (0.0)	0 (0.0)	1 (0.7)	0.183
cT2		27.7	87 (27.5)	48 (27.9)	40 (28.0)	
cT3		61.3	202 (63.9)	98 (57.0)	87 (60.8)	
cT4		10.8	27 (8.5)	26 (15.1)	15 (10.5)	
**cN-stage**						
cN0		41.7	128 (40.4)	61 (35.5)	74 (52.1)	0.011
cN1		32.6	97 (30.6)	65 (37.8)	44 (31.0)	
cN2		25.7	92 (29.0)	46 (26.7)	24 (16.9)	
**Neoadjuvant therapy**						
None		32.6	107 (34.9)	48 (27.7)	48 (33.6)	0.027
RT		31.9	89 (29.0)	72 (41.6)	38 (26.6)	
CRT		35.5	111 (36.2)	53 (30.6)	57 (39.9)	

Values are *n* (%) unless otherwise stated. LOREC, LOw REctal Cancer; L-centre, laparoscopic TME centre; R-centre, robotic TME centre; Ta-centre, transanal total mesorectal excision centre; ARJ, anorectal junction; i.q.r., interquartile range; MRF, mesorectal fascia; cT, clinical tumour; cN, clinical nodal; cM, clinical metastasis; RT, radiotherapy; CRT, chemoradiotherapy.

### Oncological outcomes

*Table 2* displays the operative, postoperative and oncological outcomes. The R1 rate of the total cohort was 6.5%. L-centres showed a trend towards superior R1 rate (CRM+ and/or DRM+) with 4.4% *versus* 8.7% in R-centres and 8.4% in Ta-centres, *P* = 0.107. Overall CRM positivity rate was 5.5%, with again comparable numbers among all expert technique centres (L-centres 3.8%, R-centres 6.9%, Ta-centres 7.7%, *P* = 0.153). Restorative procedures showed a trend towards lower CRM+ margins with 2.3% compared with 7.1%, *P* = 0.305. The DRM+ rate and incompleteness of TME were also comparable among all three technique centres.

**Table 2:** Operative and postoperative outcomes MRI-defined LOREC patients, 2015–2017

Operative and postoperative outcomes		Total *n* = 633 (%)	LOREC			
			L-centre *n* = 317	R-centre *n* = 173	Ta-centre *n* = 143	*P* value
**Technique used**						
Open		3.8	16 (5.0)	2 (1.2)	6 (4.2)	<0.001
Lap		53.0	283 (89.3)	12 (7.0)	40 (28.0)	
TaTME		17.4	12 (3.8)	1 (0.6)	97 (67.8)	
Robot		25.8	6 (1.9)	157 (91.3)	0 (0.0)	
**Procedure**						
Restorative		33.7	78 (24.6)	64 (37.2)	71 (49.7)	<0.001
Non-restorative		66.3	239 (75.4)	107 (62.8)	72 (50.3)	
Diverting stoma*						
No		40.8	30 (38.5)	17 (26.6)	40 (56.3)	<0.001
Diverting ileostomy		63.4	51 (16.1)	50 (29.1)	34 (23.8)	
End colostomy		63.5	231 (72.9)	104 (60.1)	67 (46.9)	<0.001
**Converted procedures**						
Total		4.1	16 (5.0)	4 (2.3)	6 (4.2)	0.344
Restorative		2.8	1 (1.3)	2 (3.1)	4 (5.6)	0.334
Non-restorative		4.5	15 (6.3)	2 (1.9)	2 (2.7)	
Operating time (min), median (i.q.r.)		178 (135–274)	160 (135–195)	195 (147–243)	218 (171–274)	<0.001
Intraoperative complication	Yes	5.7	16 (5.0)	12 (6.9)	8 (5.6)	0.688
Complications	CD ≥III	21.5	70 (22.1)	33 (19.2)	33 (23.1)	0.645
Anastomotic leakage*						
Total		23.9	18 (23.1)	15 (23.4)	18 (25.0)	0.942
Early (<30 days)		86.3	15 (83.3)	13 (86.7)	16 (88.9)	0.986
Late (>30 days)		13.7	3 (16.7)	2 (13.3)	2 (11.1)	
**Leak grade (ISREC)**						
A		15.7	2 (10.5)	0 (0.0)	6 (33.3)	0.026
B		49.0	13 (68.4)	8 (53.3)	5 (27.8)	
C		35.3	4 (21.1)	7 (46.7)	7 (38.9)	
Reintervention	<30 days	18.3	61 (19.2)	28 (16.3)	27 (18.9)	0.692
**Secondary stoma**						
Ileostomy		2.8	0	1 (1.6)	5 (7.0)	0.327
Colostomy		4.2	3 (3.8)	3 (4.7)	3 (4.2)	
Readmission	<30 days	15.0	46 (14.5)	25 (14.5)	24 (16.8)	0.796

**Pathological outcomes**						
R1	Total	6.5	14 (4.4)	15 (8.7)	12 (8.4)	0.107
**CRM ≤1 mm**
Total		5.5	12 (3.8)	12 (6.9)	11 (7.7)	0.153
Restorative		2.3	0	2 (3.1)	3 (4.2)	0.305
Non-restorative		7.1	12 (5.0)	10 (9.3)	8 (11.1)	
DRM ≤1 mm	Total	0.9	2 (0.6)	3 (1.7)	1 (0.7)	0.456
Incomplete TME	Total	7.4	24 (7.6)	16 (9.2)	7 (4.9)	0.337
Composite endpoint†	Total	13.1	38 (12.0)	27 (15.6)	18 (12.6)	0.514

**Oncological outcomes**						
Follow-up	months	35.3	36.0	34.6	34.7	0.374
Local recurrence	3-year	5.9	19 (6.1)	12 (6.7)	6 (4.2)	0.809
Systemic recurrence	3-year	18.8	63 (19.8)	35 (20.4)	20.9 (14.6)	0.373
Disease-free survival	3-year	73.3	236 (74.6)	120 (69.3)	108 (75.6)	0.347
Overall survival	3-year	88.3	285 (90.0)	148 (85.5)	125 (87.5)	0.436

Values are *n* (%) unless otherwise stated. *Diverting stoma and anastomotic leakage rates are calculated over the restorative procedures. †Composite endpoint consists of a positive resection margin with incompleteness of TME. LOREC, LOw REctal Cancer; L-centre, laparoscopic TME centre; R-centre, robotic TME centre; Ta-centre, transanal total mesorectal excision centre; lap, laparoscopic TME procedure; TaTME, transanal TME procedure; robot, robotic-assisted TME procedure; i.q.r., interquartile range; CD, Clavien–Dindo; ISREC, International Study Group of Rectal Cancer; R1, positive resection margin; CRM, circumferential resection margin; DRM, distal resection margin; TME, total mesorectal excision.

Median follow-up was 35.3 months with comparable 3-year local recurrence (4.2–6.7%, *P* = 0.809), systemic recurrence (14.6–20.4%, *P* = 0.373), disease-free survival (69.3–75.6%, *P* = 0.347) and overall survival (85.5–90.0%, *P* = 0.436).

Multivariable analysis for a positive circumferential resection margin (*Table 3*) showed that a threatened margin on preoperative MRI (MRF+) was the only independent risk factor (OR 4.77, *P* < 0.001).

**Table 3:** Multivariable analysis for risk on positive circumferential resection margin

CRM+		LOREC		
		OR	95% c.i.	*P* value
**Technique**				
Lap		Ref.	Ref.	Ref.
Rob				NS
TaTME				NS
Age (years)	70–80 years			NS
BMI (kg/m^2^)				NS
Sex	Male			NS
ASA	III/IV			NS
Abdominal history	Yes			NS
Distance to ARJ	0–4 cm			NS
Tumour diameter	>50 mm			NS
MRF+	≤1 mm	4.77	2.07,11.21	<0.001
cT-stage	cT4			NS
cN-stage	cN+			NS
cM-stage	cM1			NS
Neoadjuvant therapy	Yes			NS

LOREC, LOw REctal Cancer; OR, odds ratio; Lap, laparoscopic TME; Rob, robotic TME; TaTME, transanal total mesorectal excision; Ref., reference; NS, non-significant; ARJ, anorectal junction; MRF, mesorectal fasia; cT, clinical tumour; cN, clinical node; cM, clinical metastasis.

### Intraoperative and postoperative outcomes

As documented in *Table 2*, the operations used in the different centres were consistent with their expertise. In laparoscopic expert centres 89.9% of procedures were laparoscopic, 3.8% were TaTME, 1.9% were robot assisted and 5.0% were open surgeries. In robot-assisted expert centres 91.3% were robot assisted, whereas 1.2%, 0.6% and 1.2% were laparoscopic, TaTME and open procedures respectively. Finally, in TaTME expert centres 67.8% were TaTME, 28.0% were laparoscopic, 0% were robot assisted and 4.2% were open procedures.

Ta-centres had the highest rate of restorative surgery (49.7%) followed by R-TME (37.2%), with the lowest rate of restorative procedures found in L-TME centres at 24.6%, *P* < 0.001. Among the restorative patients, Ta-centres opted not to construct a temporary stoma in 56.3% of cases, compared with 9.5% in L-centres and 26.5% in R-centres, *P* = <0.001.

The overall conversion rate in the entire LOREC group was 4.1%. The conversion rate was comparable among the expert technique centres with a trend towards less conversion in R-centres at 2.3%, compared with 5.0% in L-TME and 4.2% in TaTME, *P* = 0.344. Conversion was also comparable among restorative and non-restorative procedures, *P* = 0.334.

The overall anastomotic leakage rate increased from 13.7% after 30 days to 23.9% at the end of follow-up. The majority of the early leaks were grade B or C. Comparable numbers of major complications (Clavien–Dindo ≥ III, *P* = 0.645), reinterventions (*P* = 0.692) and readmissions (*P* = 0.796) were found.

*Table 4* displays the agreed preoperative plan, the final procedure, final approach and the change in procedure or approach. In 10.4% of the patients treated in L-centres there was a change in procedural management, compared with 5.2% in R-centres and 2.1% in Ta-centres, *P* = 0.004. The majority of these changes were a change from a restorative procedure to a non-restorative procedure (5.1%), with the highest number occurring in the L-centres (9.6%) compared with R-centres (5.6%) and Ta-centres (4.2%), *P* = 0.033.

**Table 4:** Change in preoperative management plan and outcome for MRI-defined LOREC patients, 2015–2017

Change of management and outcome	LOREC				
	Total *n* = 633	L-centre *n* = 317	R-centre *n* = 173	Ta-centre *n* = 143	*P* value
**Intended procedure**					
LAR + anastomosis	236 (37.3)	95 (30.0)	68 (39.5)	73 (51.0)	<0.001
LAR + colostomy	68 (10.8)	45 (14.2)	8 (4.7)	15 (10.5)	
APR	310 (49.1)	163 (51.4)	94 (54.7)	53 (37.1)	
Unknown	18 (2.8)	14 (4.4)	2 (1.2)	2 (1.4)	
**Surgical procedure**					
LAR + anastomosis	213 (33.7)	78 (24.6)	64 (37.2)	71 (49.7)	0.001
LAR + colostomy	77 (12.2)	54 (17.0)	8 (4.7)	15 (10.5)	
APR	342 (54.1)	185 (58.4)	100 (58.1)	57 (39.9)	
**Change in procedural plan**					
No	587 (92.9)	284 (89.6)	163 (94.8)	140 (97.9)	0.004
Yes	45 (7.1)	33 (10.4)	9 (5.2)	3 (2.1)	
**Change in procedure**					
TEM -> LAR	–	–	–	–	1.000
Non-rest -> Rest	3 (0.5)	1 (0.3)	2 (1.2)	–	0.287
Non-rest -> Non-rest	10 (1.6)	9 (2.8)	1 (0.6)	–	0.134
−Hartmann -> APE	8 (1.3)	7 (2.2)	1 (0.6)	–	
−APE -> Hartmann	2 (0.3)	2(0.6)	–	–	
Rest -> Non-rest	32 (5.1)	23 (7.3)*	6 (3.5)	3 (2.1)*	0.033
−Hartmann	12 (1.9)	11 (3.5)	1 (0.6)	0 (0.0)	
−APE	20 (3.2)	12 (3.8)	5 (2.9)	3 (2.1)	
**Cause of change in plan^†^**					
Low tumour	12 (28.6)	5 (15.6)	6 (85.7)	1 (33)	
Stapling difficulty	18 (42.9)	18 (56.3)	–	–	
Functional outcome	3 (7.1)	3 (9.4)	–	–	
Vascularization	1 (2.4)	–	–	1 (33)	
Unclear	6 (14.2)	5 (15.6)	–	1 (33)	
Other	2 (4.8)	1 (3.1)	1 (14.3)	–	

Values are *n* (%). *Difference is significant between L-centre and Ta-centre. ^†^Cause of change of operative plan only given for change from restorative to non-restorative surgery and non-restorative to other non-restorative procedure. LOREC, LOw REctal Cancer; L-centre, laparoscopic TME centre; R-centre, robotic TME centre; Ta-centre, transanal total mesorectal excision centre; LAR, low anterior resection; APE, abdominal perineal excision; TEM, transanal endoscopic microsurgery; rest, restorative; non-rest, non-restorative.

The main reason for change in procedural management was stapling difficulty (*n* = 18, 42.9%), which was only found in L-centres. Other reasons for a change in management were: low lying tumour (*n* = 12), expected poor functional outcome (*n* = 3), vascularization problems of the bowel (*n* = 1), peritoneal depositions in Douglas (*n* = 1) and tearing of staple row (*n* = 1). Distance to the ARJ ≤1 cm (OR 0.25 (95% c.i. 0.083 to 0.59), *P* = 0.005) and L-TME were independent risk factors for change in the preoperative plan (L-TME: ref; R-TME: OR 0.34 (95% c.i. 0.12 to 0.77), *P* = 0.024; TaTME: OR 0.17 (95% c.i. 0.027 to 0.59), *P* = 0.021), see *Table 5*.

**Table 5:** Multivariable analysis change of preoperative procedural plan

Change of plan		LOREC OR (95% c.i.)
Sex	Male	NS
ASA	III/IV	NS
History of abdominal surgery	Yes	NS
Distance to ARJ (MRI)	≤1 cm	0.25 (0.083,0.59) *P* = 0.005
MRF involvement	≤1 mm	NS
cT4	Yes	NS
cM+	Yes	NS
**Neoadjuvant therapy**		
RTx		NS
CRT		NS
**Technique**		
Lap		Ref.
Rob		0.34 (0.12,0.77) *P* = 0.024
TaTME		0.17 (0.027,0.59) *P* = 0.021

LOREC, LOw REctal Cancer; OR, odds ratio; NS, non-significant; ARJ, anorectal junction; MRI, magnetic resonance imaging; MRF, mesorectal fascia; cT, clinical tumour stage; cM, clinical metastasis stage; RTx, radiotherapy; CRT, chemoradiotherapy; Lap, laparoscopic TME, Rob, robotic-assisted TME; TaTME, transanal total mesorectal excision.

## Discussion

This observational, retrospective, multicentre cohort study aimed to evaluate the use of laparoscopic, robot-assisted and transanal minimally invasive techniques in 11 expert centres for LOREC based on the MRI-defined LOREC definition. The main finding is that high-quality oncological outcomes can be achieved with all three techniques, even in LOREC. Additionally, although conversion rate was comparable between the groups, the rate of change of intended procedure was significantly higher in the L-centres.

The comparable and high-quality oncological outcomes align with the findings of a recent meta-analysis which showed comparable outcomes in oncologic efficacy for all three minimally invasive techniques^25^. The CRM+ rates observed in this study are favourable when compared with results from recent RCTs comparing minimally invasive techniques for rectal cancer, considering both LOREC and non-LOREC patients^4,26–29^. For instance, the COLOR (COlon cancer Laparoscopic or Open Resection) II-trial, which analysed tumours in the low rectum (<5 cm), separately found CRM+ rates of 9% in L-TME and 22% in open TME^28^. However, a more recent Dutch LOREC study with a similar cut-off of 5 cm from the anal verge found a CRM+ rate of 5.2%^30^. In the latter study, the mesorectal fascia was less often threatened (29.2% *versus* 34.5%), a known risk factor for a positive resection margin, as also shown in the current multivariable analysis, *Table 4*^30,31^. The higher rate of CRM+ in the non-restorative cases is also consistent with contemporary literature^30,32^.

Interestingly, in R-centres and Ta-centres restorative surgery rates were higher (37.2% and 49.7%) when compared with L-centres (24.6%). Thus it seems that with the newer techniques the creation of an anastomosis is attempted more often. This is also seen in the recently published REAL trial comparing L-TME with R-TME. The fabrication of a low anastomosis is known to be more at risk for anastomotic leakage^33^. The anastomotic leakage rate among the three expert technique centres was spread evenly among the restorative procedures per expert technique centre, averaging 23.9%. Although this appears very high, anastomotic leak rates of around 20% are common when including late leakages (beyond 30 days)^34–36^.

While lower conversion rates might have been expected with R-TME or TaTME, these were actually comparable and most likely reflect the expertise of surgeons beyond their learning curve^37^. An interesting finding in the present study is the analysis of change of preoperative procedural plan during the procedure, which can be interpreted as a different type of conversion of the operation^38^. The majority of the procedural changes were observed in the L-TME group followed by R-TME and TaTME surgery, *P* = 0.004. The majority (70%) of these L-TME changes of plan were from a restorative procedure to a non-restorative procedure. The main reason for a change of management was difficulty with distal stapling, which was only reported for L-TME. The use of the Transanal rectal Transection with a Single Stapled (TTSS) anastomosis technique avoids distal stapling, even in L-TME and R-TME, by enabling a single stapled double pursestring anastomosis via a transanal approach without the need for a full transanal TME dissection^39^. However, low location of the tumour was found in all three techniques and could be a consequence of pushing the boundaries of the technique and/or misjudgment of the resection margins before surgery. This, despite improvements in diagnostic imaging, still remains one of the main reasons for changes in perioperative decision-making^40^.

Unfortunately, as is the case with a lot of retrospective reports, important information about quality of life after TME surgery is lacking^41,42^. However, a study in this field and a systematic review both showed that patients value not having a stoma in combination with being cured of cancer, whilst avoiding complications, as the most important factors in colorectal surgical care treatment^43,44^. It can be hypothesized that the impact of a change of preoperative management into an unexpected permanent stoma is even greater. For this reason, when encountering a LOREC patient, referral to an expert R-centre or Ta-centre can be considered.

The strength of this study lies in the ability to compare all three minimally invasive techniques side by side, utilizing a standardized MRI-based, reproducible classification method for LOREC. It is unique in describing the preoperative intent and how this differs from the final procedure. Several limitations should also be mentioned. Selection bias is of potential concern because of the retrospective collection of all operative cases (including non-dedicated procedures) from selected expert centres in The Netherlands. However, in multivariable analysis we corrected for the dedicated technique used. Although the entire cohort is of reasonable size, the group sizes per technique differ. This might have caused some factors not showing significance despite absolute differences between groups.

With all three minimally invasive TME techniques a high-quality oncological resection can be achieved in the treatment of MRI-defined LOREC. Higher rates of non-restorative procedures were observed in L-centres, and patients are prone to an intraoperative change in the surgical plan. For patients with LOREC with an explicit wish for a restorative procedure, referral to an experienced robotic or Ta-centre could be considered.

## Data Availability

The data that support the findings of this study are available upon request from the corresponding author, R.H. The data are not publicly available due to information that could compromise the privacy of research participants, however most information is also accessible from the DCRA (Dutch ColoRectal Audit) upon request.
